# Improved Tolerance of Mycorrhizal *Torreya grandis* Seedlings to Sulfuric Acid Rain Related to Phosphorus and Zinc Contents in Shoots

**DOI:** 10.3390/jof7040296

**Published:** 2021-04-14

**Authors:** Lina Xia, Changliang Shao, Naili Zhang, Aiping Wu, Jiangbo Xie, Yajing Qiu, Xiaobin He, Jia Pei, Xudong Wang, Yanhong Wang

**Affiliations:** 1 State Key Laboratory of Subtropical Silviculture, Zhejiang A & F University, Hangzhou 311300, China; 2019102032013@stu.zafu.edu.cn (L.X.); ecologyxjb@zafu.edu.cn (J.X.); qiuyajing202103@163.com (Y.Q.); 2019602041013@stu.zafu.edu.cn (X.H.); 2019602042032@stu.zafu.edu.cn (J.P.); wangxd@zafu.edu.cn (X.W.); 2 Institute of Agricultural Resources and Regional Planning, Chinese Academy of Agricultural Sciences, Beijing 100081, China; shaochangliang@caas.cn; 3 College of Forestry, Beijing Forestry University, Beijing 100083, China; zhangnaili@bjfu.edu.cn; 4 Hunan Provincial Key Laboratory of Rural Ecosystem Health in Dongting Lake Area, Ecology Department, College of Resources and Environment, Hunan Agricultural University, Changsha 410128, China; wuaip@hunau.edu.cn

**Keywords:** acid rain, arbuscular mycorrhizal fungus, acid-tolerance index, nutritional quality, mycorrhizal growth response

## Abstract

Acid rain (AR) is an increasingly serious environmental problem that frequently occurs in Southern China with sulfuric acid rain (SAR) as the main type. SAR can negatively affect the growth and physiological properties of trees, but mycorrhizal associations may mitigate such detrimental effects. However, the mechanisms by which arbuscular mycorrhizal fungi control SAR-induced impacts on *Torreya grandis* plants remain unclear. A pot experiment was conducted on *T. grandis* seedlings, an economically important tree species in Southern China, in which inoculated and non-inoculated *T. grandis* seedlings were subjected to three simulated SAR regimes (pH of 5.6, 4.0, and 2.5, respectively) to examine the effects on the growth, osmotic regulation, and nutrient absorption of these seedlings. The results show that, although SAR had no effect on the accumulation of biomass, it significantly decreased the concentrations of proline and soluble protein, shoot Zn^2+^, P, K^+^, and Ca^2+^ concentrations, and the Fe^2+^ and Mn^2+^ concentrations of shoots and roots. Mycorrhizal inoculation, especially with *Rhizophagus irregularis*, significantly increased total biomass, proline concentration, and the Zn^2+^, P, and K^+^ concentrations in the shoots of *T. grandis* under lower pH conditions. Moreover, our findings suggest that the combination of root colonization, acid tolerance, and the concentrations of shoot-P, shoot-Zn^2+^, and root-Fe^2+^ of *T. grandis* jointly conferred mycorrhizal benefits on the plants under SAR conditions. Given the enhancement of the nutritional quality of *T. grandis* owing to mycorrhizal associations, inoculation with *R. irregularis* may be preferable for the culturing and management of these plants under acidic conditions.

## 1. Introduction

Acid rain (AR) is mainly derived from the drastic emissions of sulfur dioxide (SO_2_) and nitrogen oxides (NO_X_) and is usually known as a type of rainwater with a pH < 5.6. Due to this low pH, AR poses serious environmental hazards worldwide [1]. In the past 20 years, the centers of AR have transferred from Europe and North America to East Asia, especially India and China; presently, approximately one-fifth of Chinese cities suffer from AR [2]. However, the area over which AR falls in China is continuously increasing with the acceleration of urbanization and industrialization [3,4]. Sulfuric acid rain (SAR), the main type of AR, is particularly prevalent in Southern China, where the mean pH of rainfall in recent years has often been less than 4.6 and has even fallen to 3.6 in some extreme cases [5,6,7,8].

Previous studies have shown that SAR can induce soil and water acidification, forest decline, and damage to buildings, resulting in heavy economic losses to local governments [9,10]. Furthermore, SAR has complex ecological consequences in plants, such as stunting their height, degrading root system growth, interrupting the accumulation of low-molecular-weight solutes, and obstructing the transportation of nutrients, which can adversely influence biomass accumulation, decrease plant resistance to unfavorable conditions, and induce mortality [11,12,13]. Therefore, physical, chemical, and/or biological approaches applied to remediate the detrimental effects of SAR or improve resistance to SAR have received increased attention [12,14,15,16,17].

Arbuscular mycorrhizal fungi (AMF), an important component of soil biota, occur naturally in acidic soils [18,19,20,21]. Through symbiotic association, fungal partners obtain shelter and fixed carbon from the host plants and in return provide multiple benefits to these plants, such as improved uptake of mineral nutrients, especially phosphorus and other relatively immobile micronutrients such as zinc, magnesium, copper, and calcium, reduced oxidative stress, and maintained water balance, which can positively impact plant growth and improve resistance to abiotic stresses [22,23,24,25]. Although AMF may be adversely affected by SAR [20,26,27], some studies have reported enhanced growth and performance of mycorrhizal plants under SAR conditions [28,29,30]. For example, mycorrhizal inoculation positively affects the growth and nutrient uptake of *Sorghum bicolor* [30], *Calamagrostis villosa* [31], *Koelreuteria paniculata* [32], and *Thuja occidentalis* [28], whereas it has little to no effect on the growth of *Deschampsia flexuosa* [31] under simulated SAR. Furthermore, the beneficial effects of *Acaulospora tuberculata* were higher than those of *Glomus fistulosum* and *G. mosseae* [31] under acidic conditions. These responses indicate that the efficacies of AMF on plants under acidic conditions are controversial and appear to depend on the fungal and host plant species. To date, most related studies have been conducted in crops or herbaceous plants, and the mechanisms underlying the protective effects of AMF on tree species under SAR remain poorly understood.

Chinese torreya (*Torreya grandis* Fort. ex Lindl. cv. Merrillii) is an economically important tree species that has been used in food and traditional medicine for more than 1000 years in Southeastern China [33,34]. Zhejiang Province, the main planting region of *T. grandis*, suffers from the most frequent SAR, with pH values varying from 5.6 to 3.8 [7]. *T. grandis* has been reported to form mycorrhizal associations with several AMF taxa [35]. However, this tree species is moderately sensitive to SAR, and the preferred pH value is approximately 4.5 [36]. In this study, we hypothesized that AMF colonization could mediate SAR-induced effects on *T. grandis* with enhanced nutrient uptake. To test this, a greenhouse experiment was conducted to explore the effects of AMF on the growth, osmotic adjustment, and nutrient absorption of *T. grandis* seedlings under simulated SAR regimes. The results of this study should improve our understanding of the mechanisms underlying the alleviation of SAR-induced impacts on plants via mycorrhizal inoculation.

## 2. Materials and Methods

### 2.1. Plant Materials and AMF Preparation

The seeds of *T. grandis* were obtained from the Hualong Nursery Corporation (Hangzhou, China). On 1 March 2017, the seeds were surface-sterilized with a 5% sodium hypochlorite solution (Yonghua Chemical Co., Ltd., Changshu, China) for 15 min and then rinsed with deionized water. The seeds were then sown in trays containing 1 kg of autoclaved substrate with a mixture of sand and peat (1:1, *v*/*v*) in a growth chamber set to 20 °C and 15 °C during the day and night, with 16- and 8-h photoperiods, respectively. After emergence, 90 seedlings of identical sizes were selected for use in the following treatments.

Two dominant AMF species in the field, *Rhizophagus irregularis* (N.C. Schenck and G.S. Sm.) C. Walker and A. Schüßler (BGC BJ09) and *Funneliformis mosseae* (T.H. Nicolson and Gerd.) C. Walker and A. Schüßler (BGC HUN03B), were selected as the inocula, which were supplied by the Bank of Glomeromycota in China at the Institute of Plant Nutrients and Resources, Beijing Municipal Academy of Agriculture and Forestry Science. *R. irregularis* was initially isolated from the rhizosphere of tomatoes in Langfang (Hebei Province, China) and *F. mosseae* was isolated from the rhizosphere of *Roegneria kamoji* Ohwi in Chenzhou (Hunan Province, China). These two mycorrhizal inocula were multiplied for five months using *Sorghum bicolor* L. by the trap culture method in a plastic pot with fine sand as the substrate [37]. In this experiment, the *R. irregularis* inoculum contained ~200 spores g^−1^ and abundant mycelia, whereas the *F. mosseae* inoculum contained ~160 spores g^−1^, as well as colonized roots and mycelium fragments. Both fungal inocula were in accordance with the highest possible number test [38].

### 2.2. Experimental Design

On 14 April 2017, the seedlings were moved into a greenhouse situated at the Pingshan Research Station of Zhejiang A & F University in Hangzhou city, Zhejiang Province, China (30°15′ N, 119°43′ E). After six days of adaptation to the greenhouse environment, 72 healthy and similar-sized seedlings were transplanted into plastic pots (16.5 cm × 18 cm × 12 cm) containing 2 kg of soil substrate. Single seedlings were planted in each pot. The soil substrate used in this experiment consisted of a mixture of field soil and peat in the ratio 3:1 (*v*/*v*) with γ-irradiation (25 kGy) [39]. The substrate had the following properties: pH 5.6, organic matter 22.8 mg·g^−1^, total N 0.85 mg·g^−1^, and available P 0.42 mg·g^−1^.

The experiment was performed using 12 factorial combinations of SAR and AMF regimes. The three intensities of SAR regimes employed were pH 5.6, 4.0, and 2.5. The AMF regimes comprised four levels: autoclaved non-mycorrhizal inoculum, inoculated with *R. irregularis*, inoculated with *F. mosseae*, and inoculated with the combination of *R. irregularis* and *F. mosseae*. Six replicate pots were set for each treatment combination, for a total of 72 pots. The amount of inoculum for single-species inoculation was 40 g per pot, and the amount of each AMF species for non-mycorrhizal and two-species inoculation was 20 g per pot. The inocula were placed into 15-cm pots just below the roots of the seedlings after transplanting [40]. Meanwhile, the pots with non-mycorrhizal inoculation were supplied with a 5 mL aqueous filtrate of the two fungal species (non-sterilized AMF mixture-distilled water ratio = 1:3) filtered twice through Whatman No. 1 paper to correct for possible differences in soil microbial populations [41].

On 18 June 2017, simulated SAR treatments were explored. In this study, we selected pH values of 5.6, 4.0, and 2.5 as the treatment regimes, which were consistent with the annual changes in pH values for SAR in most areas of Southern China [7]. We first prepared a stock acid solution by mixing 0.5 mol L^−1^ of H_2_SO_4_ and 0.5 mol L^−1^ of HNO_3_ (Shanghai Lingfeng Chemical Co. Ltd., Shanghai, China) at a 2:1 ratio, which was equivalent to the ionic ratio of natural precipitation at the local site [7]. Second, the corresponding SAR regimes were generated by adding distilled water (average pH: ~6.8) to the stock acid solution. A total of 13.49 L of simulated SAR was applied to each pot with 253 mL applied every two days, which is approximately equivalent to the annual precipitation in Hangzhou city, Zhejiang Province, China [42]. Lastly, 5 mL of adjusted Hoagland solution was received weekly by each pot [43]. These experiments were conducted from 20 April to 25 November 2017. At the end of the experiment, the shoots and roots of seedlings were harvested separately.

### 2.3. Measurement of Plant Biomass and AMF Colonization

All plants were divided into roots, stems, and leaves after the experiment ended, and the biomass of each part was weighed after drying at 60 °C for 48 h. Mycorrhizal colonization was determined by first randomly selecting the root segments of three plants from each treatment for digestion in 10% KOH (Xilong Chemical Co. Ltd., Guangzhou, China) at 90 °C for 90 min, followed by rinsing with distilled water and then acidification in 2% HCl (Shanghai Lingfeng Chemical Co. Ltd., Shanghai, China) at ambient temperature for 5 min. The root segments were then stained with 0.05% trypan blue (Sangon Biotech Co. Ltd., Shanghai, China) at 90 °C for 1 h in a water bath as described by Phillips and Hayman [44]. Finally, the stained root segments were examined under a microscope according to the gridline intercept method [45].

### 2.4. Measurement of Proline and Soluble Protein Contents

After harvesting, the proline and soluble protein contents of the leaves of four plants randomly selected from each treatment were immediately determined via spectrophotometry using commercial kits (Nanjing Jiancheng Institute of Bioengineering, Nanjing, China) [46].

### 2.5. Measurement of Plant Nutrient Contents

The dried shoot and root samples of three randomly selected plants from each treatment were ground separately, and their macro- and micronutrient contents were analyzed. The N and P contents were determined using the Kjeldahl and ammonium molybdate blue methods, respectively [47], whereas the K^+^, Na^+^, Ca^2+^, Mg^2+^, Fe^2+^, Zn^2+^, and Mn^2+^ contents were measured following the methods described by Colla et al. [48] using an atomic absorption spectrophotometer (AA7000; Shimadzu, Japan).

### 2.6. Data Analysis

To quantify the influence of mycorrhizal colonization, the mycorrhizal growth response (MGR) was calculated as described by Johnson et al. [49]:

(1) $$\mathrm{MGR}=log_{e}\left(\frac{DW_{\mathrm{AMF}}}{Avg(DW_{non-\mathrm{AMF}})}\right)$$

where *DW*_AMF_ is the total dry weight of mycorrhizal plants and $Avg{(\mathrm{DW}}_{non-\mathrm{AMF}})$ is the mean dry weight of non-colonized plants subjected to identical pH conditions (*n* = 6).

The acid−tolerance index (ATI) of each plant was computed following He et al. [29]:

(2) $$\mathrm{ATI}=1-\frac{B_{i,max}-B_{i}}{B_{i,max}}$$

where *B_i_*_,*max*_ is the largest total biomass of the six plants at pH 5.6, with identical AMF inoculation, and *B_i_* is the total biomass of each of the six plants at pH levels of 2.5 and 4.0 (both are the stressful pH values in our study). Target species for which 0 < ATI < 0.3, 0.3 < ATI < 0.6, and ATI > 0.6, were considered as acid-sensitive, moderately acid-sensitive, and acid-tolerant, respectively.

A two−way analysis of variance was used to study the effects of AMF and SAR on plant growth, physiochemical parameters, MGR, ATI, and nutrient contents. Before analysis, all data were subjected to Levene’s test for the equality of variance and the Shapiro–Wilk test for normality. When the interactive effects of SAR and AMF were significant (*p* < 0.05), a least significant difference test was used to compare the significance of plant parameters among different treatments. Pearson’s correlation analysis was also used to analyze the relationship between paired traits of *T. grandis*, and linear regression was used to determine the relationship between MGR and related parameters. Statistical analyses were conducted using SPSS v. 23.0 (SPSS Inc., Chicago, IL, USA) and R v. 4.0.2 [50], and graphs were generated using Origin 2018 (Origin Lab Co., Northampton, MA, USA).

## 3. Results

### 3.1. Mycorrhizal Colonization

**Figure 1:**
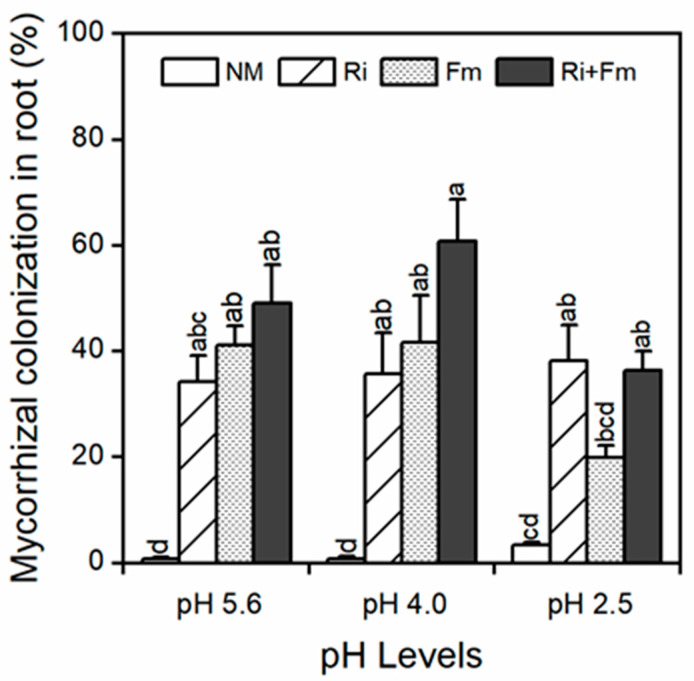
Effects of arbuscular mycorrhizal fungi (AMF) on mycorrhizal colonization of *Torreya grandis* under acid rain with pH 5.6, 4.0, and 2.5. NM, Ri, Fm, and Ri+Fm represent the four AMF treatments: inoculation with no mycorrhizal fungi, with *Rhizophagus irregularis,* with *Funneliformis mosseae*, and with the combination of the two fungi inoculum, respectively. Values are presented as the mean ± SE (*n* = 3). Different letters indicate a significant difference (*p* < 0.05).

As shown in Figure 1, there was nearly no mycorrhizal colonization (MC) in the roots of *T. grandis* seedlings with non-mycorrhizal inoculation (MC < 3.35%). Meanwhile, three treatments of AMF species successfully colonized the roots of *T. grandis* at all pH levels, with root colonization ranging from 19.9 to 60.83%. There were no significant differences in MC between different AMF across all pH levels and the highest root colonization was recorded for plants inoculated with a combination of *R. irregularis* and *F. mosseae* at higher pH levels (5.6 and 4.0), and with *R. irregularis* at pH 2.5. Significant interactive effects of SAR and AMF on MC were observed (Table S1).

### 3.2. Plant Growth and Physiochemical Parameters

**Figure 2:**
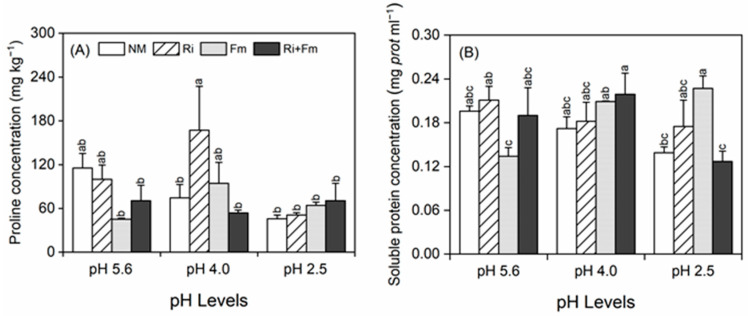
Effects of arbuscular mycorrhizal fungi on total dry weight (**A**) and root:shoot ratio (**B**) of *Torreya grandis* under acid rain with pH 5.6, 4.0, and 2.5. NM, Ri, Fm, and Ri+Fm represent the four AMF treatments: inoculation with no mycorrhizal fungi, with *Rhizophagus irregularis*, with *Funneliformis mosseae*, and with the combination of the two fungi inoculum, respectively. Values are presented as the mean ± SE (*n* = 6). Different letters indicate a significant difference (*p* < 0.05).

In this study, SAR did not significantly affect the total dry weight (TDW) of *T. grandis* seedlings and, at pH 4.0, the TDWs of mycorrhizal plants were higher than those of non-mycorrhizal plants, especially in bi-inoculated plants. At pH 2.5, the highest TDW was found in plants inoculated with *R. irregularis* (Figure 2A). Additionally, significant interactive effects were detected between SAR and AMF on TDW (Table S1). Moreover, neither SAR nor AMF had significant impacts on the distribution of biomass (Figure 2B; Table S1). These responses showed that mycorrhizal inoculation mainly affected the biomass accumulation, not the biomass allocation, of *T. grandis* under acidic conditions.

**Figure 3:**
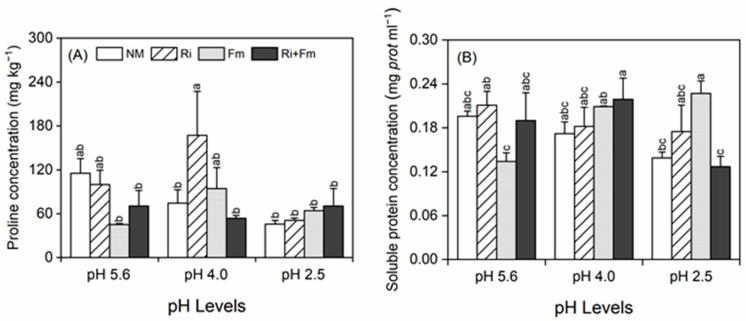
Effects of arbuscular mycorrhizal fungi on proline concentration (**A**) and soluble protein concentration (**B**) of *Torreya grandis* under acid rain with pH 5.6, 4.0, and 2.5. NM, Ri, Fm, and Ri+Fm represent the four AMF treatments: inoculation with no mycorrhizal fungi, with *Rhizophagus irregularis,* with *Funneliformis mosseae*, and with the combination of the two fungi inoculum, respectively. Values are presented as the mean ± SE (*n* = 4). Different letters indicate a significant difference (*p* < 0.05).

Different changes in proline concentrations were observed between mycorrhizal and non-mycorrhizal plants in response to SAR; at pH 4.0 and 2.5, the proline concentrations were higher in mycorrhizal plants, among which plants inoculated with *R. irregularis* and the combination of *R. irregularis* and *F. mosseae* possessed the highest proline content records at pH 4.0 and pH 2.5, respectively (Figure 3A). SAR alone had no effect on the soluble protein (SP) concentrations of *T. grandis* seedlings (Table S1). At pH 4.0, the highest SP concentration was recorded in bi-inoculated plants, whereas at pH 2.5, the highest concentration was recorded in plants inoculated with *F. mosseae* alone, with concentrations 62.8% higher than those of non-mycorrhizal plants (Figure 3B). A significant interaction between SAR and AMF was found in the SP concentrations of the plants (Table S1).

### 3.3. Concentrations of Shoot and Root Mineral Nutrients

**Figure 4:**
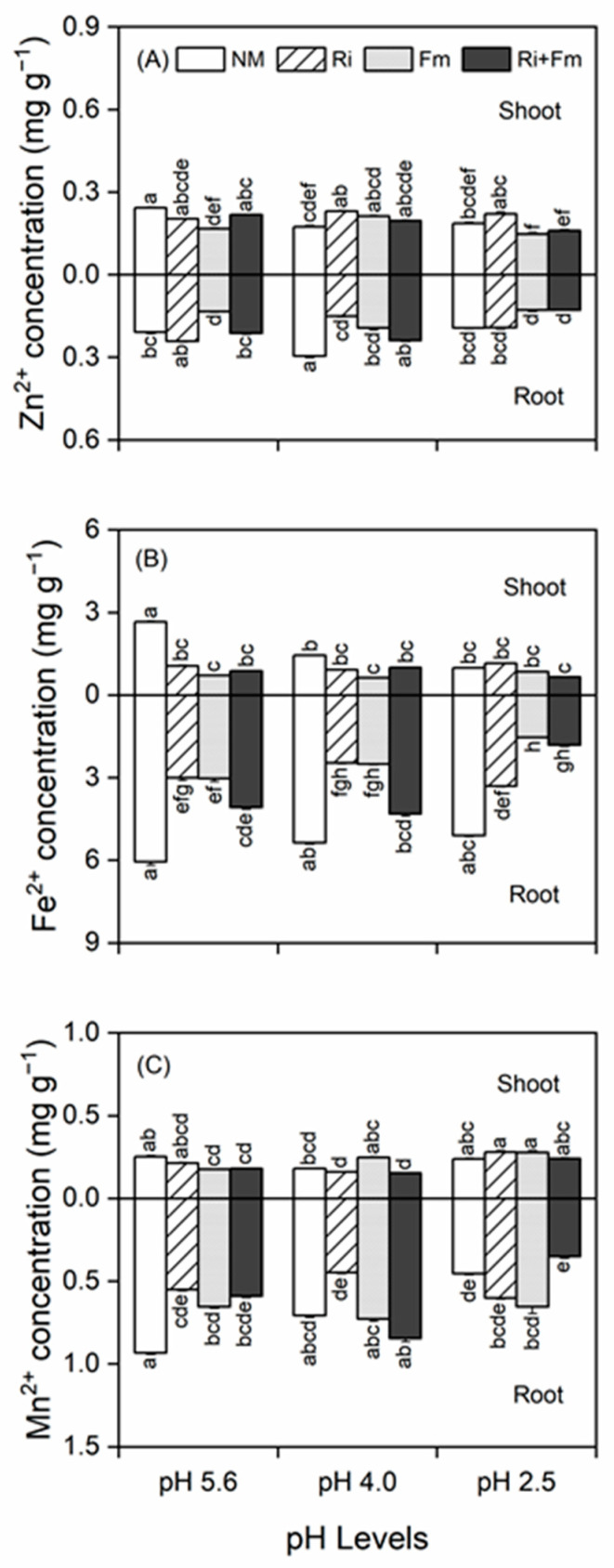
Effects of arbuscular mycorrhizal fungi on micronutrients of Zn^2+^ (**A**), Fe^2+^ (**B**), and Mn^2+^ (**C**) in shoot and root of *Torreya grandis* under acid rain with pH 5.6, 4.0, and 2.5. NM, Ri, Fm, and Ri+Fm represent the four AMF treatments: inoculation with no mycorrhizal fungi, with *Rhizophagus irregularis,* with *Funneliformis mosseae*, and with the combination of the two fungi inoculum, respectively. Values are presented as the mean ± SE (*n* = 3). Different letters indicate a significant difference (*p* < 0.05).

SAR alone significantly decreased the concentrations of micronutrients (Zn^2+^, Fe^2+^, and Mn^2+^) in *T. grandis* seedlings. At lower pH levels (4.0 and 2.5), the shoot Zn^2+^ concentrations of mycorrhizal plants were higher than those of non-mycorrhizal plants, especially in plants inoculated with *R. irregularis*; however, the root Zn^2+^ concentrations of these plants were lower than those of non-mycorrhizal plants (Figure 4A). At all pH levels, AMF inoculation significantly decreased the shoot and root Fe^2+^ concentrations compared with their respective controls (Figure 4B). Similarly, at a pH of 5.6, the shoot and root Mn^2+^ concentrations in mycorrhizal plants were lower than those of non-mycorrhizal plants, whereas at lower pH levels, there were no differences between them with either one of the AMF inocula (Figure 4C). SAR, AMF inoculation, and their interactions significantly impacted the Zn^2+^, Fe^2+^, and Mn^2+^ concentrations in the shoots and roots of *T. grandis* seedlings (Table S1).

**Figure 5:**
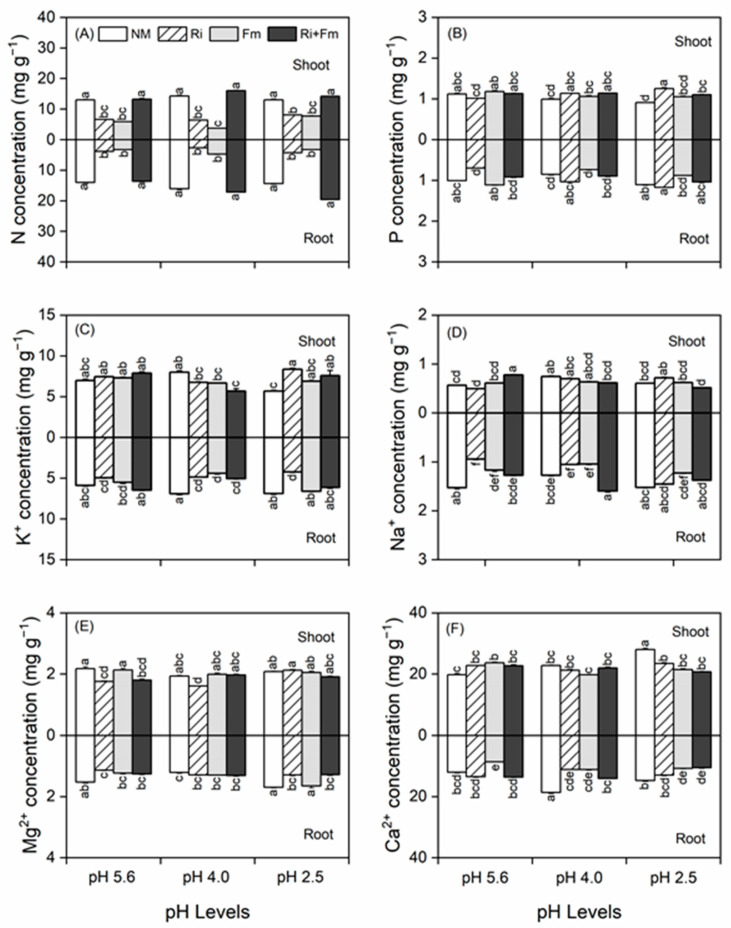
Effects of arbuscular mycorrhizal fungi on macronutrients of N (**A**), P (**B**), K^+^ (**C**), Na^+^ (**D**), Mg^2+^ (**E**), and Ca^2+^ (**F**) in shoot and root of *Torreya grandis* under acid rain with pH 5.6, 4.0, and 2.5. NM, Ri, Fm, and Ri+Fm represent the four AMF treatments: inoculation with no mycorrhizal fungi, with *Rhizophagus irregularis,* with *Funneliformis mosseae*, and with the combination of the two fungi inoculum, respectively. Values are presented as the mean ± SE (*n* = 3). Different letters indicate a significant difference (*p* < 0.05).

The concentrations of N, P, K^+^, Na^+^, Ca^2+^, and Mg^2+^ in the shoots and roots of *T. grandis* seedlings were significantly affected by SAR, AMF, and their interactions (Table S2). SAR alone did not have a significant effect on N concentrations in the shoots and roots of non-mycorrhizal or mycorrhizal plants and, with the exception of the bi-inoculated plants, the N concentrations in the shoots and roots of mycorrhizal plants were lower than those of non-mycorrhizal plants (Figure 5A). SAR significantly decreased P concentrations in the shoots of non-mycorrhizal plants, but not in mycorrhizal plants. At the lowest pH (=2.5), AMF significantly increased the P concentrations in shoots, especially with inoculation by *R. irregularis* (Figure 5B). The P concentrations in shoots were dominantly affected by SAR and AMF, whereas those in the roots were less affected. SAR alone negatively affected the K^+^ concentrations in the shoots of non-mycorrhizal plants, but not in the roots of any plants (Figure 5C). At a pH of 5.6, the K^+^ concentrations in the shoots of mycorrhizal plants were lower than those of non-mycorrhizal plants at pH 4.0 but higher than those of their counterparts; at all pH values, the K^+^ concentrations in the roots of mycorrhizal plants were lower than those of non-mycorrhizal plants (Figure 5C).

The effects of SAR and AMF on Na^+^ and Mg^2+^ concentrations in the shoots and roots varied (Figure 5D,E). The Mg^2+^ and Ca^2+^ concentrations in the shoots were consistently higher than those in the roots (Figure 5E,F). SAR significantly increased the Ca^2+^ concentrations in the shoots of non-mycorrhizal plants but not in mycorrhizal plants, whereas those in the roots initially increased and then frequently declined (Figure 5F). At the lowest pH level (2.5), the Ca^2+^ concentrations in the shoots and roots of mycorrhizal plants were lower than those of non-mycorrhizal plants.

### 3.4. Mycorrhizal Benefits and the Relationships between Plant Traits

**Figure 6:**
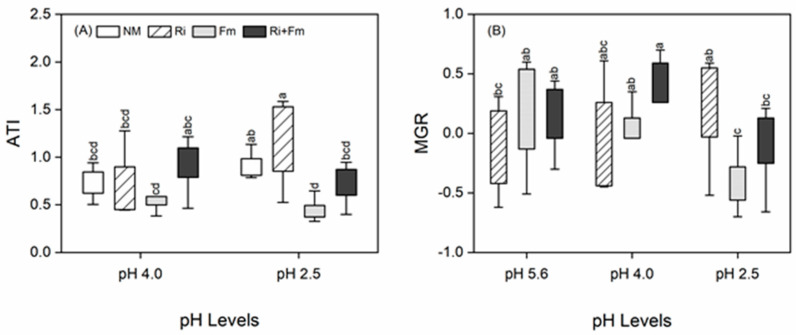
Effects of arbuscular mycorrhizal fungi on acid−tolerance index (ATI) (**A**) and mycorrhizal growth response (MGR) (**B**) of *Torreya grandis* under acid rain with pH 5.6, 4.0, and 2.5. NM, Ri, Fm, and Ri+Fm represent the four AMF treatments: inoculation with no mycorrhizal fungi, with *Rhizophagus irregularis,* with *Funneliformis mosseae*, and with the combination of the two fungi inoculum, respectively. Values are presented as the mean ± SE (*n* = 6). Different letters indicate a significant difference (*p* < 0.05).

**Figure 7:**
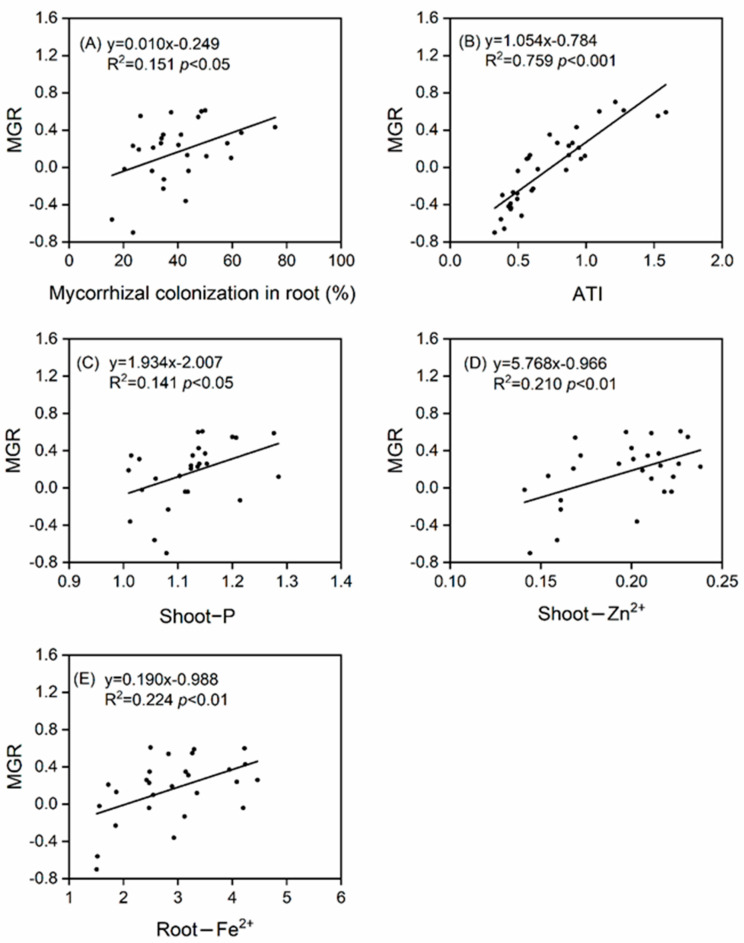
Relationships between mycorrhizal growth response (MGR) and mycorrhizal colonization (**A**), acid−tolerance index (ATI) (**B**), shoot−P (**C**), shoot−Zn^2+^ (**D**), and root−Fe^2+^ (**E**) of *Torreya grandis* under the interactions of arbuscular mycorrhizal fungi and acid rain.

At lower pH values (4.0 and 2.5), the ATI values suggested that non-mycorrhizal plants, and those inoculated with *R. irregularis* alone or with the combination of *R. irregularis* and *F. mosseae*, were tolerant to SAR (ATI > 0.6), whereas plants inoculated only with *F. mosseae* were moderately acid-sensitive (0.3 < ATI < 0.6). At a pH of 2.5, the highest ATI value was detected in plants inoculated with *R. irregularis*, which was 20.2, 157.6, and 67.3% higher than that in non-mycorrhizal plants, plants inoculated with *F. mosseae* alone, and plants inoculated with the combination of the two AMF species, respectively (Figure 6A). With decreases in pH, the MGR of plants inoculated with *R. irregularis* increased, whereas those inoculated with *F. mosseae* or with both AMF species decreased significantly, suggesting that *R. irregularis* more positively impacted plant growth, especially at pH 2.5 (Figure 6A). Furthermore, MGR was positively and linearly correlated with MC, ATI, shoot-P, shoot-Zn^2+^, and root-Fe^2+^ concentrations, which may be the mechanism underlying mycorrhizal efficacy (Figure S2, Figure 7).

## 4. Discussion

In this study, we aimed to explore the effects of AMF on the growth, physicochemical parameters, and nutrient uptake of *T. grandis* under simulated sulfuric acid rain. We found that SAR alone negatively affected certain physicochemical parameters and nutrient acquisition, and AMF colonization greatly increased total dry biomass, proline concentration, and P, Zn^2+^, and K^+^ concentrations in the shoots of *T. grandis*, especially with inoculation by *R. irregularis* under the lower pH levels when compared to the non-mycorrhizal controls. These results support our hypothesis that AMF can, to some degree, mediate the unfavorable effects of SAR on *T. grandis* seedlings. Furthermore, our results show that mycorrhizal efficiency was mainly enhanced at lower pH levels, implying the specific control of SAR.

Biomass can be a useful indicator of plant growth and performance under SAR stress [16]. In this study, SAR alone had no effect on the total biomass of *T. grandis*, which is consistent with a study on maize grown under pH values ranging from 2.0 to 7.0 [51]. The response of biomass to SAR suggested that SAR did not overstress the growth of *T. grandis*, which could be ascribed to the relatively high acid-tolerance of most *T. grandis* plants, with the exception of those inoculated with *F. mosseae* (Figure 6A). However, AMF inoculation significantly increased the biomass accumulation of *T. grandis* under lower pH conditions (i.e., 4.0 and 2.5), which is consistent with the observations of *C. villosa* under acidic conditions [31]. Previous studies have also shown similar mycorrhizal efficiencies for 24 tropical forage legumes grown in a pH of 4.36 [52], cassava grown in soil with pH 3.9, and sorghum in pH 4.5 [53]. However, enhancements to biomass were not found in the mycorrhizal plants of *D. flexuosa* grown under SAR conditions [31]. Thus, mycorrhizal benefits depend on both the host plant species and pH levels. Moreover, AMF inoculation in this study mainly affected the accumulation of biomass rather than its allocation, which agrees with the response of *Chrysanthemum morifolium* to mycorrhizal inoculation under salinity stress [37].

AMF inoculation not only enhanced the growth of *T. grandis*, but also the acquisition of elements in this study. In our study, mycorrhizal efficiency was found to be directly correlated to P and Zn^2+^ concentrations in shoots and Fe^2+^ concentration in roots. P is an important macronutrient in plant cells, including the sugar-phosphates that facilitate respiration and photosynthesis, the phospholipids that make up plant membranes, and the nucleotides used in metabolism and in DNA and RNA. Meanwhile, a deficiency in P stunts plant growth, resulting in dark green coloration, necrotic spots on the leaves, slender stems, and delayed maturation [54]. P is the most commonly reported mineral nutrient to be enhanced with mycorrhizal inoculation under stressful conditions [23,26,55], which was corroborated in our study but was not noted in previous studies on mycorrhizal sorghum grown in acid soils [30]. Zinc is an essential micronutrient for chlorophyll biosynthesis in some plants, and Zn deficiencies are characterized by reductions in intermodal growth and by small and distorted leaves with white necrotic spots. Additionally, Zn in food can increase human appetites and promote neurological development, thereby benefiting human health [54]. In the present study, the mycorrhizal efficiency of Zn^2+^ in plant shoots is in agreement with maize grown on acid soils [26] and sorghum grown in soil at pH 4.1 [53]. Fe^2+^ is another nutritional element and is normally less available at higher pH values [53], whereas AMF inoculation did not offer benefits, which was in line with the results on *Stylosanthes guianensis* [56] but was contrary to results on sorghum grown in acidic soils [30]. Notably, in this study, *R. irregularis* was more efficient at increasing P and Zn^2+^ acquisition in *T. grandis* shoots than other mycorrhizal fungi, which could therefore enhance the yield and nutritional quality of *T. grandis* nuts in SAR-impacted areas. Numerous studies have confirmed the positive effects of AMF on nutrient uptake and plant quality, which can be considered as an assurance against their deterioration caused by unfavorable factors [23,57,58].

Improved P and Zn^2+^ uptake by AMF under acidic conditions in this study might have been due to increased absorption by the extensive hyphal network for exploring larger soil volumes and increasing root growth, decreasing the distance that the elements had to diffuse to reach plant roots, or by the release of organic acids and phosphatase to chemically modify organic compounds by hyphae [53,59]. Moreover, the results of this study reveal that the percentage of root colonization in mycorrhizal plants is not affected by SAR with pH values ranging from 2.5 to 5.6. This is a finding that is consistent with the root colonization of spring oats (*Avena sativa* L.) and potatoes (*Solanum tuberosum* L.) at soil pH values between 4.5 and 7.5. Nevertheless, the highest colonization of any of the three fungal types in this experiment occurred at pH 4.0. Reportedly, the optimal pH values for the maximal root colonization of cassava (*Manihot esculenta* Crantz) with *G. manihotis*, *Acaulospora mellea*, and *Entrophospora colombiana* are 4.4, 4.5, and 4.8, respectively [26]. Evidently, AMF differ in their pH preferences; however, in this study, mycorrhizal efficiency was linearly correlated with root colonization, which is contrary to the finding that the benefits of AMF on the growth of fenugreek (*Trigonella foenum*-*graecum*) were independent of root colonization under salinity stress [60]. Furthermore, AMF performed differently in terms of mediating the effects of SAR, and the plants benefited more from inoculation with *R. irregularis* under lower pH regimes. These functional differences among AMF species may reflect stress-specific adaptive mechanisms [61,62].

It remains unclear how exactly AMF promote plant performance under unfavorable conditions. Previous studies have suggested that the improvement in nutrient uptake (especially P), increased osmotic protective agents, such as proline, soluble sugars, or amino acids, and/or the enhancement of physiological processes, such as photosynthesis or water absorption capacity, are responsible for the mycorrhizal benefits [23,63,64]. In our study, a combination of root colonization, acid tolerance, and the concentrations of shoot-P, shoot-Zn^2+^, and root-Fe^2+^ in *T. grandis* likely worked together to confer mycorrhizal benefits under SAR.

## 5. Conclusions

In this study, AMF inoculation, especially with *R. irregularis*, significantly increased total biomass, proline content, and the Zn^2+^, P, and K^+^ concentrations in the shoots of *T. grandis* under high SAR intensities, providing evidence that AMF can mediate SAR-induced negative effects on *T. grandis* plants to a certain extent. Furthermore, our study revealed that the combination of root colonization, acid tolerance, and the concentrations of shoot-P, shoot-Zn^2+^, and root-Fe^2+^ in *T. grandis* may be responsible for mycorrhizal efficiency under SAR. In fact, with improvements in living standards, consumer interest in the quality of edible products, such as the nuts of *T. grandis*, has increased. Considering the specific mycorrhizal efficacy of AMF on shoot P and Zn^2+^ contents in response to SAR observed here, inoculation with *R. irregularis* may be a preferable means of enhancing the nutritional quality of *T. grandis* grown in regions suffering from frequent SAR. However, our experiment was conducted under greenhouse conditions, and mycorrhizal efficacy can be affected by several environmental factors. Therefore, field trials are now required to verify our conclusions.

## Data Availability

Data is contained within the article and Supplementary Materials.

## Supplementary Materials

The following are available online at https://www.mdpi.com/article/10.3390/jof7040296/s1, Table S1: Effects of sulfuric acid rain (SAR), arbuscular mycorrhizal fungi (AMF), and their interactions on growth, physiological, and biochemical indexes, and micronutrient concentrations of *Torreya grandis* seedlings; Table S2: Effects of sulfuric acid rain (SAR), arbuscular mycorrhizal fungi (AMF), and their interactions on macronutrient concentrations of *Torreya grandis* seedlings; Figure S1: Colonization of arbuscular mycorrhizal fungi in the roots of *Torreya grandis* plants under sulfuric acid rain; Figure S2: Correlation coefficients of the variables of *Torreya grandis* across the twelve treatment combinations. Blue and red colors indicate positive and negative significant correlations, respectively. In the upper triangle, the darker the circle color is, the stronger the correlation is. Significance levels: * *p* < 0.05; ** *p* < 0.01; *** *p* < 0.001.

## Abbreviations

AR	acid rain
SAR	sulfuric acid rain
AMF	arbuscular mycorrhizal fungi
MGR	mycorrhizal growth response
ATI	acid-tolerance index
MC	mycorrhizal colonization
TDW	total dry weight
SP	soluble protein
