# Supplementary figures and images for: Improved Tolerance of Mycorrhizal Torreya grandis Seedlings to Sulfuric Acid Rain Related to Phosphorus and Zinc Contents in Shoots
Source: J Fungi (Basel). 2021 Apr 14;7(4):296. doi: 10.3390/jof7040296 (PMC8070988; doi:10.3390/jof7040296)

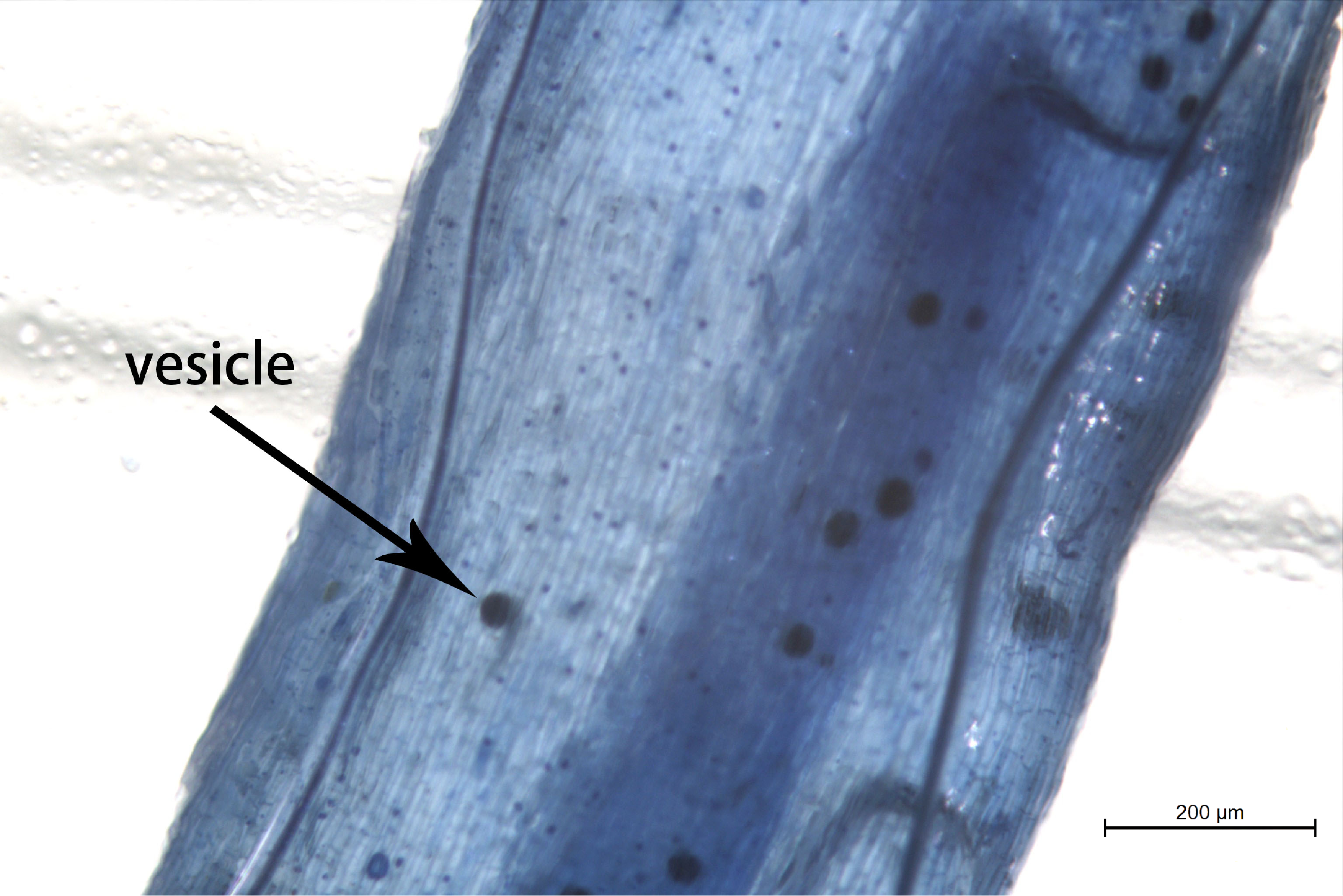

Supplement: Supplementary file 1 [file jof-07-00296-s001.zip › jof-1159691-si revised/Figure S1..tif]

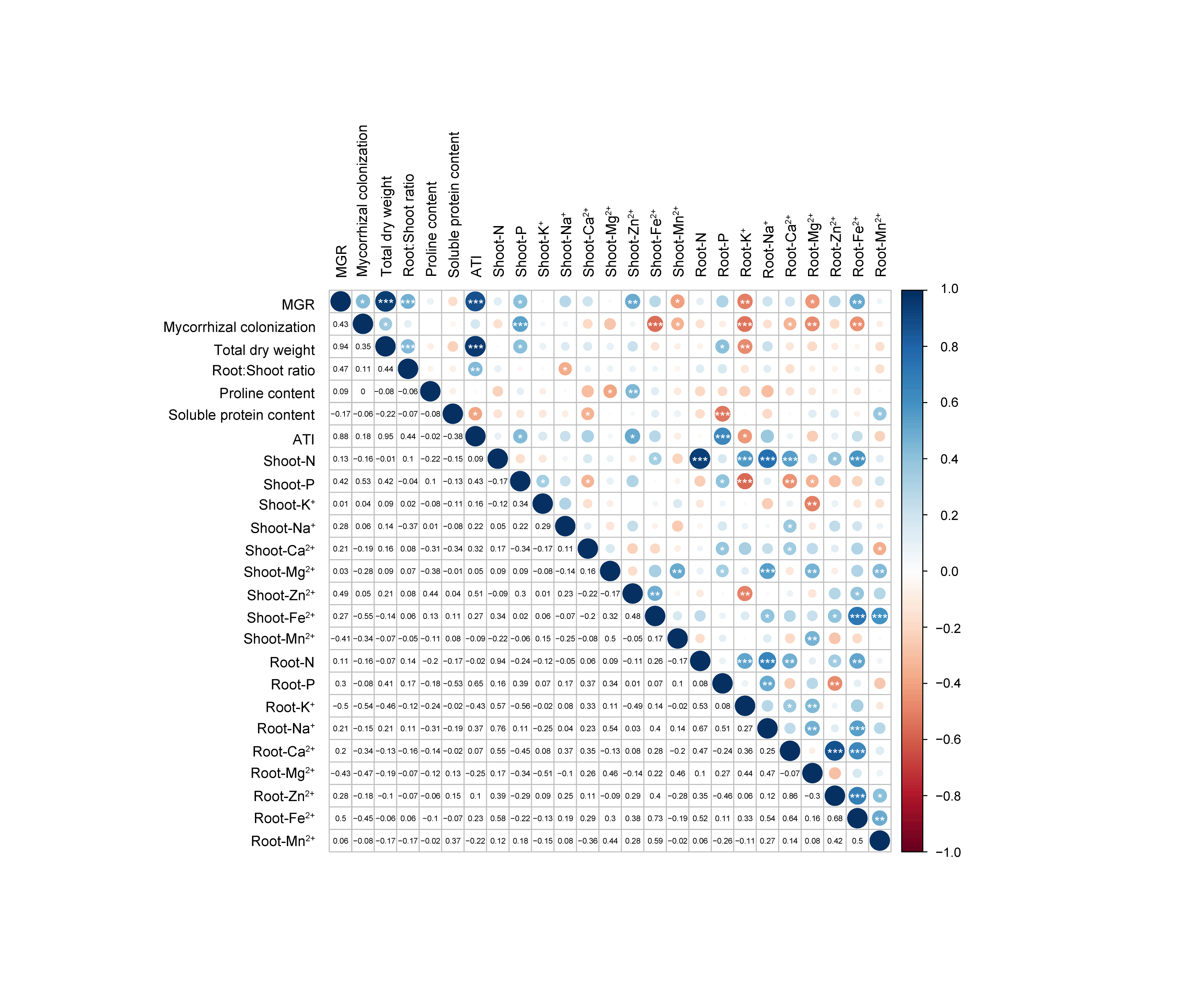

Supplement: Supplementary file 1 [file jof-07-00296-s001.zip › jof-1159691-si revised/Figure S2..tif]
