# Supplementary material for: Improved Tolerance of Mycorrhizal Torreya grandis Seedlings to Sulfuric Acid Rain Related to Phosphorus and Zinc Contents in Shoots
Source: J Fungi (Basel). 2021 Apr 14;7(4):296. doi: 10.3390/jof7040296 (PMC8070988; doi:10.3390/jof7040296)
Supplement: Supplementary file 1 [file jof-07-00296-s001.zip › jof-1159691-si revised/Table S1..doc]

**Table S1.** Effects of sulfuric acid rain (SAR), arbuscular mycorrhizal fungi (AMF) and their interactions on growth, physiology and biochemistry index, and micronutrients content of *Torreya grandis* seedlings.

| Plant index | SAR | AMF | SAR × AMF |
| --- | --- | --- | --- |
| Mycorrhizal colonization in root (%) | 1.811^ns^ (2,24) | 101.430^***^ (3,24) | 3.469^**^ (6,24) |
| Total dry weight (g) | 2.097^ns^ (2,58) | 3.404^*^ (3,58) | 2.657^*^ (6,58) |
| Root:shoot ratio (g g^-1^) | 0.952^ns^ (2,58) | 1.384^ns^ (3,58) | 0.474^ns^ (6,58) |
| Proline concentration (mg kg^-1^) | 6.180^**^ (2,36) | 0.240^ns^ (3,36) | 2.263^ns^ (6,36) |
| Soluble protein concentration (mg *prot* ml^-1^) | 0.737^ns^ (2,35) | 4.007^*^ (3,35) | 3.397^**^ (6,35) |
| Shoot-Zn^2+^ (mg g^-1^) | 13.520^***^ (2,24) | 75.030^***^ (3,24) | 59.243^***^ (6,24) |
| Shoot-Fe^2+^ (mg g^-1^) | 55.963^***^ (2,24) | 175.521^***^ (3,24) | 826.402^***^ (6,24) |
| Shoot-Mn^2+^ (mg g^-1^) | 114.930^**^ (2,24) | 16.835^***^ (3,24) | 43.181^***^ (6,24) |
| Root-Zn^2+^ (mg g^-1^) | 106.135^***^ (2,24) | 68.166^***^ (3,24) | 103.588^***^ (6,24) |
| Root-Fe^2+^ (mg g^-1^) | 38.611^***^ (2,24) | 571.671^***^ (3,24) | 116.876^***^ (6,24) |
| Root-Mn^2+^ (mg g^-1^) | 24.214^***^ (2,24) | 76.263^***^ (3,24) | 117.257^***^ (6,24) |
| Acid-tolerance index (ATI) | 0.552^ns^ (1,38) | 7.383^***^ (3,38) | 3.048^*^ (3,38) |
| Mycorrhizal growth response (MGR) | 3.954^*^ (2,43) | 3.368^*^ (2,43) | 2.569^ns^ (4,43) |

Data in the table are expressed as *F*-values and followed by *df* values in parentheses.

Significance levels: ^ns^ *P* > 0.05; ^*^ *P* < 0.05; ^**^ *P* < 0.01; ^***^ *P* < 0.001.
