# Supplementary material for: Improved Tolerance of Mycorrhizal Torreya grandis Seedlings to Sulfuric Acid Rain Related to Phosphorus and Zinc Contents in Shoots
Source: J Fungi (Basel). 2021 Apr 14;7(4):296. doi: 10.3390/jof7040296 (PMC8070988; doi:10.3390/jof7040296)
Supplement: Supplementary file 1 [file jof-07-00296-s001.zip › jof-1159691-si revised/Table S2..doc]

**Table S2.** Effects of sulfuric acid rain (SAR), arbuscular mycorrhizal fungi (AMF) and their interactions on macronutrients content of *Torreya grandis* seedlings.

| Plant index | SAR | AMF | SAR × AMF |
| --- | --- | --- | --- |
| Shoot-N (mg g^-1^) | 25.008^***^ (2,24) | 337.762^***^ (3,24) | 60.981^***^ (6,24) |
| Shoot-P (mg g^-1^) | 43.893^***^ (2,24） | 62.507^***^ (3,24) | 30.993^***^ (6,24) |
| Shoot-K^+^ (mg g^-1^) | 11.686^***^ (2,24) | 23.619^***^ (3,24) | 19.200^***^ (6,24) |
| Shoot-Na^+^ (mg g^-1^) | 96.047^***^ (2,24) | 43.535^***^ (3,24) | 76.802^***^ (6,24) |
| Shoot-Ca^2+^ (mg g^-1^) | 12.989^***^ (2,24) | 107.981^***^ (3,24) | 66.351^***^ (6,24) |
| Shoot-Mg^2+^ (mg g^-1^) | 60.522^***^ (2,24) | 7.398^**^ (3,24) | 20.278^***^ (6,24) |
| Root-N (mg g^-1^) | 23.999^***^ (2,24) | 2159.492^***^ (3,24) | 93.459^***^ (6,24) |
| Root-P (mg g^-1^) | 174.543^***^ (2,24) | 45.563^***^ (3,24) | 86.030^***^ (6,24) |
| Root-K^+^ (mg g^-1^) | 16.511^***^ (2,24) | 158.295^***^ (3,24) | 67.586^***^ (6,24) |
| Root-Na^+^ (mg g^-1^) | 198.312^***^ (2,24) | 44.250^***^ (3,24) | 87.062^***^ (6,24) |
| Root-Ca^2+^ (mg g^-1^) | 30.991^***^ (2,24) | 83.819^***^ (3,24) | 99.647^***^ (6,24) |
| Root-Mg^2+^ (mg g^-1^) | 8.410^**^ (2,24) | 54.758^**^ (3,24) | 25.078^***^ (6,24) |

Data in the table are expressed as *F*-values and followed by *df* values in parentheses.

Significance levels: ^ns^ *p* > 0.05; ^*^ *p* < 0.05; ^**^ *p* < 0.01; ^***^ *p* < 0.001.
